# Pasos Hacia La Salud II: study protocol for a randomized controlled trial of a theory- and technology-enhanced physical activity intervention for Latina women, compared to the original intervention

**DOI:** 10.1186/s13063-022-06575-4

**Published:** 2022-08-01

**Authors:** Andrea S. Mendoza-Vasconez, Tanya Benitez, Shira Dunsiger, Kim M. Gans, Sheri J. Hartman, Sarah E. Linke, Britta A. Larsen, Dorothy Pekmezi, Bess H. Marcus

**Affiliations:** 1 Department of Behavioral and Social Sciences, Brown University School of Public Health, Providence, USA; 2 Human Development and Family Sciences, College of Liberal Arts and Sciences, University of Connecticut, Storrs, USA; 3 Herbert Wertheim School of Public Health and Human Longevity Science, University of California San Diego, La Jolla, USA; 4 Department of Health Behavior, The University of Alabama at Birmingham School of Public Health, Birmingham, USA

**Keywords:** Hispanic, Latina, Female, eHealth, mHealth, Technology, Exercise, Physical activity, Health promotion, Randomized controlled trial

## Abstract

**Background:**

Latinas are at increased risk for many lifestyle-related chronic diseases and are one of the least physically active populations in the US Innovative strategies are needed to help Latinas achieve the health benefits associated with physical activity (PA). This manuscript describes the study protocol of the Pasos Hacia La Salud II Study, which builds upon our previous research to test an enhanced individually-tailored, text-message and website-delivered, Spanish-language intervention (enhanced intervention), in comparison to the original web-based Pasos Hacia La Salud Intervention (original intervention).

**Methods:**

Sedentary Latinas between the ages of 18–65 will be recruited and will complete an orientation and baseline assessments. Participants will be subsequently randomized to the original intervention, or the Enhanced Intervention, which has greater targeting of theoretical constructs such as self-efficacy, enjoyment, and social support, and which uses text messages and more dynamic and refined website features to encourage increased website use. Using a linear mixed effects regression model, we will simultaneously estimate the intervention effects on mean accelerometer-measured hours/week of moderate-to-vigorous PA (MVPA) at 6, 12, 18, and 24 months, with a subject-specific intercept (intent-to-treat sample). Change in self-reported MVPA, measured via the 7-day Physical Activity Recall, will be assessed as a secondary outcome using a similar model. We will investigate potential mediators of the intervention effect using a multiple mediation approach, and potential moderators by evaluating potential interactions. As an exploratory outcome, we will study the differences (among both study arms) in cost, in US dollars, per minute increases in weekly mean MVPA.

**Discussion:**

The original Pasos PA intervention showed efficacy in helping Latinas increase PA; we expect the Enhanced Intervention to help a larger proportion of participants to increase and maintain their PA long term. This web- and text-based enhanced intervention could have great reach and dissemination potential, which could be capitalized on in the future to help to advance health equity. Adaptations made in response to the COVID-19 pandemic are also described in this manuscript.

**Trial registration:**

Clinical Trial Number: NCT03491592. First posted April 9, 2018.

## Administrative information

Note: the numbers in curly brackets in this protocol refer to SPIRIT checklist item numbers. The order of the items has been modified to group similar items (see http://www.equator-network.org/reporting-guidelines/spirit-2013-statement-defining-standard-protocol-items-for-clinical-trials/).

**Table:** 

Title {1}	SPIRIT guidance: Descriptive title identifying the study design, population, interventions, and, if applicable, trial acronym. **Pasos Hacia La Salud II: Study Protocol for a Randomized Controlled Trial of a Theory- and Technology-Enhanced Physical Activity Intervention for Latina Women, Compared to the Original Intervention.**
Trial registration {2a and 2b}.	SPIRIT guidance: Trial identifier and registry name. If not yet registered, name of intended registry. Item 2b is met if the register used for registration collects all items from the World Health Organization Trial Registration Data Set. **Clinical Trial Number: NCT03491592**
Protocol version {3}	Protocol # (#1708001868). Most recently approved amendment: Amendment # 9, August 11, 2021
Funding {4}	SPIRIT guidance: Sources and types of financial, material, and other support. **National Institutes of Health (NIH)/National Cancer Institute (NCI)** **Grant Number: 2R01CA159954**
Author details {5a}	SPIRIT guidance: Affiliations of protocol contributors. Andrea S. Mendoza-Vasconez, andrea_mendoza@brown.edu^1^ Tanya Benitez tanya_benitez@brown.edu^1^ Shira Dunsiger shira_dunsiger@brown.edu^1^ Kim M. Gans Kim_Gans@brown.edu^1, 2^ Sheri J. Hartman, sjhartman@health.ucsd.edu^3^ Sarah E. Linke slinke@health.ucsd.edu^3^ Britta A. Larsen blarsen@health.ucsd.edu^3^ Dorothy Pekmezi, dpekmezi@uab.edu^4^ Bess H. Marcus, bess_marcus@brown.edu^1^ 1 Department of Behavioral and Social Sciences, Brown University School of Public Health 2 Human Development and Family Sciences, College of Liberal Arts and Sciences, University of Connecticut 3 Dep Herbert Wertheim School of Public Health and Human Longevity Science, University of California, San Diego 4 Department of Health Behavior, The University of Alabama at Birmingham School of Public Health
Name and contact information for the trial sponsor {5b}	SPIRIT guidance: Name and contact information for the trial sponsor. National Cancer Institute of the National Institutes of Health
Role of sponsor {5c}	SPIRIT guidance: Role of study sponsor and funders, if any, in study design; collection, management, analysis, and interpretation of data; writing of the report; and the decision to submit the report for publication, including whether they will have ultimate authority over any of these activities. The sponsor was not involved in study design, data collection/management, data analysis and interpretation, report writing, or the decision to submit the report for publication.

## Introduction

### Background and rationale {6a}

Almost two-thirds of Latinas are insufficiently active [1], putting them at increased risk for many chronic diseases. Furthermore, Latinos are the largest ethnic minority group in the USA [2] and experience disproportionally high rates of many chronic diseases such as obesity and diabetes [3, 4]. Despite the benefits of physical activity (PA) for enhancing health, reducing illness, disability, and early death, Latinas are one of the least active populations in the USA. Only 45.0% of Latinas in the USA meet the national guidelines for aerobic PA (≥150 min/week of at least moderate-intensity aerobic activity) [5], compared to 51.9% of non-Latino White women and 53.9% of Latino men [6]. Moreover, Latinas report numerous social, environmental, economic, linguistic, and cultural factors that limit their access to PA programs and resources [4]. Thus, innovative strategies are needed to overcome these barriers and help Latinas achieve the health benefits associated with PA.

Using technology to improve the accessibility of health care interventions shows great promise for providing health promotion programs to hard-to-reach populations at low cost. Internet usage has grown rapidly among Latinos in the past decade [7, 8], yet to our knowledge, there are few PA interventions using this mode of delivery in Latina populations. Building on the success of our research team’s evidence-based print intervention, and the rapid growth of Internet use among Latinos [9], we conducted formative research with Latinas regarding their Internet use behaviors to develop an Internet-based PA [9]. Results of our intervention showed that participants achieved significant PA improvements at 6 and 12 months [10, 11]; however, participants were on average still well below national PA guidelines, suggesting that a more intensive Internet-based approach may be necessary to help sedentary Latinas reach and sustain health-enhancing PA levels.

In addition to needing more intensive approaches to support increasing PA, longer follow-up is needed to understand maintenance of behavior over time. Most PA intervention studies with Latinas have not followed participants beyond 12 months. The few studies that have examined longer-term PA maintenance (i.e., 24 months) in Latino populations have focused on samples of older adults [12] those diagnosed with type 2 diabetes [13], and parent-child dyads [14]; however, none have targeted the general population of Latina adults. Maintenance of behavior change is necessary to have an enduring impact on reducing risk of chronic diseases.

The current study addresses these two important gaps in the literature. This study is one of the first to use a combined text message and website-based intervention to enhance the reach and dissemination potential of PA interventions in Latinas. Although Internet and text messaging-based interventions are common generally, they are still rarely used with Latinas, despite their now equal access to Internet and cell phone technology [8]. Second, there has been little research conducted on long-term maintenance of PA in the general population and this will be the first study to examine 24-month PA intervention outcomes among Latinas. It will also move the field forward by recruiting Spanish-speaking Latinas, a group reporting high rates of inactivity and related health disparities and less access to traditional English-only PA resources and programs. The use of culturally appropriate PA interventions that address the unique sociocultural factors that influence PA among Latinas is essential to reducing the excess burden of illness, disability, and early death in one of the fastest-growing populations in the USA [2].

### Objectives {7}

This study builds upon our previous intervention and formative research with Latinas in order to test an individually-tailored, text-message and web-based, Spanish-language intervention (enhanced intervention), in comparison to the original web-based Pasos Hacia La Salud Intervention (original intervention). This manuscript describes the study protocol of the Pasos Hacia La Salud II Study. The primary aims of the study are to (1) test the efficacy of the enhanced intervention for increasing minutes of moderate-to-vigorous PA (MVPA), compared to the original intervention, and (2) test the efficacy of the enhanced intervention for PA maintenance at 24 months, compared to the original intervention. Additional questions of interest include (1) evaluating whether the theoretically driven constructs (e.g., self-efficacy, enjoyment, social support) mediate the intervention effect; (2) exploring potential moderators of efficacy including acculturation, health literacy, and BMI; and (3) evaluating the cost-effectiveness of delivering the original vs. the enhanced intervention.

### Trial design {8}

We are using a superiority randomized controlled trial (RCT) with an approximately equal (1:1) allocation ratio to test the relative efficacy of the original evidence-based theory-driven, PA Internet intervention for Latinas vs. an enhanced version of this intervention. The study was designed to test changes in MVPA at the end of the active intervention period (6 months) and MVPA maintenance over time (12- and 24-month follow-up), to examine changes in theoretical constructs as they relate to PA initiation and maintenance, and to determine the cost-effectiveness of both intervention arms. Both the original and the enhanced Internet-based interventions are guided by social cognitive theory (SCT) [15] and the transtheoretical model (TTM) [16].

## Methods: Participants, interventions, and outcomes

### Study setting {9}

The study is being conducted at Brown University in Providence, Rhode Island, USA.

### Eligibility criteria {10}

Potential participants are self-identified Latinas between the ages of 18–65 years who are sedentary, defined as engaging in less than 60 min/week of MVPA. Eligibility includes the ability to read, write and speak in Spanish (screened by the Short Test of Functional Literacy in Adults [17, 18] at baseline) and having regular access to a text-message compatible cell phone and to an Internet-connected device through home, work, or their community (e.g., public library, community center, neighbor’s house). Exclusion criteria include a history of coronary heart disease (history of myocardial infarction or symptoms of angina), stroke, orthopedic conditions which limit mobility, or any other serious medical condition that would make PA unsafe (based on the modified Physical Activity Readiness Questionnaire) [19], hospitalization due to a psychiatric disorder in the past 3 years, BMI above 45 kg/m^2^, current or planned pregnancy, and/or moving from the area within 2 years.

### Who will take informed consent? {26a}

During an initial orientation session, bilingual/bicultural staff provide a presentation delineating study requirements and rights of participation, outline potential risks and benefits, and answer potential participants’ questions and/or concerns about the study. At the end of the session, Latinas who are interested and eligible to participate in the study complete the informed consent process.

### Additional consent provisions for collection and use of participant data and biological specimens {26b}

N/A. No biological specimens were collected as part of this study, and no special provisions were included in the consent regarding the collection and use of participant data.

## Interventions

### Explanation for the choice of comparators {6b}

Results of our prior technology-based PA intervention, Pasos Hacia La Salud, showed that participants achieved significant PA improvements [10]; however, participants were on average still well below national PA guidelines of 150 min of MVPA per week [5], suggesting that a more intensive approach may be necessary to help sedentary Latinas reach and sustain health-enhancing PA levels. In the present study, we thus are randomizing individuals to one of two Spanish language, individually tailored Internet-based PA interventions: (1) the original Pasos Hacia La Salud web-based Intervention and (2) an Enhanced Pasos Hacia La Salud Intervention based on results and participant feedback from previous studies. The enhanced intervention adds text messages that encourage participants to log in to the website with greater frequency and stay on longer, and more dynamic, refined website features as well as greater targeting of self-efficacy, enjoyment, and social support. We will test whether these enhanced features and continued use of the website throughout the maintenance phase lead to greater increases in MVPA and better long-term maintenance than the original intervention.

### Intervention description {11a}

#### Original intervention

The original Internet-based program is an individually tailored Spanish language intervention, guided by SCT [15] and the TTM [16]. Participants randomized to the original intervention condition have an in-person baseline session to conduct a goal-setting session and orientation to the intervention website. An interventionist first meets with the participants to guide them through setting their own personalized MVPA goal and teach them how to gradually build up to meeting national guidelines. The goal-setting session is based on principles of motivational interviewing, and also teaches problem-solving skills so participants can adjust plans when necessary. In addition to this baseline goal-setting session, participants complete goal-setting sessions at 6, 12, 18, and 24 months.

Participants are then shown how to log into the website and use all the web tools for increasing their MVPA, including (1) calendars for entering MVPA goals and logging weekly activity, (2) questionnaires to be filled out each month to generate tailored content (see below), (3) a discussion board where participants can interact, (4) information about local PA resources and places to be active, and (5) tips for overcoming common barriers. Participants are encouraged by staff to regularly access the website and receive email prompts to visit the website and complete online surveys. Emails are sent weekly during month 1, every other week during months 2 and 3, and monthly during months 4–12.

Participants receive individually tailored website intervention content based on their responses to online questionnaires. Tailored content includes a feedback report (available through the website monthly for the first 12 months of the study) that draws from a bank of over 300 paragraphs addressing different psychosocial and environmental factors affecting PA, including self-efficacy and social support. Feedback reports cover six themes: (1) current stage of motivational readiness for PA according to the TTM; (2) how to increase their self-efficacy; (3) their use of cognitive and behavioral strategies associated with PA; (4) how the participant compares to individuals who are physically active and with national guidelines (normative feedback); (5) how the participant compares to her prior responses (progress feedback); and (6) information about PA, such as health benefits, how to monitor heart rate, or how to properly warm up. Additionally, depending on participant responses to questionnaires, they receive access to an online PA information manual. Manuals provide information matched to that individual’s stage of motivational readiness for regular PA; those in the early stages receive more information on the basic benefits of PA and how to take the first steps, while those doing regular PA receive more information on strategies for maintenance.

In addition to the website and emails, participants receive 3 brief phone calls (5–15 min each) at 1 week, 1 month, and 9 months to answer questions about using the website and support participants in revising PA goals. Participants continue to have access to the website in months 13–24, but do not receive additional emails or contacts from the study.

#### Enhanced intervention

The participants in the enhanced intervention arm receive all the intervention components of the original intervention described above plus: (1) text messages and adaptive goal setting to drive participants back to the website and keep them there longer, and (2) data-driven, technology supported enhanced intervention content targeting theoretical constructs that influenced PA behavior in Latinas from the original study. Additionally, participants in the enhanced intervention arm continue to receive a tapered dose of study materials (i.e., reports, text messages, and calls) during the second year of the study. Details of the enhanced intervention are provided below:

##### Increasing website use

Data from our previous trials [20] indicated that success was associated with a larger website “dose” (i.e., more logins, longer time on the website, and greater frequency of use of the dynamic website features). Thus, enhancements to the original intervention are aimed at encouraging participants to increase their use of the website and its interactive features. Participants receive two text messages per week during the first 6 months of the study, followed by once weekly texts during months 7–12, and biweekly texts during months 13–24. Texts (in Spanish) provide brief tailored feedback on website use (e.g., “Congrats, you logged on to the website today. Keep it up!”, “You haven’t logged into the website yet this week. Click here to check out the new blog post on Zumba!”), along with reminders to use the tracking features on the website (e.g., “Remember to log your daily activity on the website’s activity calendar so you can keep track of your progress and compare with others in the study!”). Participants have the opportunity to earn points towards non-monetary incentives (e.g., water bottles) for regularly logging on to the website and using interactive features (e.g., posting a goal, completing a questionnaire; features associated with the greatest PA change in the original study). Additionally, a virtual "medal" is awarded to participants in weeks that they register at least 150 min of activity; medals earned are displaced in the goal-setting tab. Winning the medal also places participants in the 150 Minute Club, and their name is posted on the 150 Minute Club board. Any enhanced intervention arm participant can view the names of new and recurring members of the 150 Minute Club.

To encourage participants to use the website more frequently, the PA tracking feature has been refined for adaptive goal setting. While the original intervention emphasizes a relatively static goal of reaching national guidelines of 150 min of MVPA per week, the goal setting feature for the enhanced intervention encourages participants to increase their current PA by approximately 20% each month (e.g., “Over the past month, you’ve been reporting about 100 minutes of moderate intensity PA per week on average. Are you ready to bump that up? Let’s shoot for 120 minutes per week now. See the tip sheets for ideas on how to add 20 more minutes!”).

##### Further targeting key theoretical constructs and participant feedback

Tip sheets are posted on the Enhanced website that provides more in-depth information on constructs shown in our previous trials to be key intervention targets, including self-efficacy, social support, and enjoyment. Additional tip sheets are posted biweekly during the first 6 months of the study, monthly during months 7–12, and once during months 14, 16, 18, and 21.

To further promote self-efficacy and enjoyment, text messages provide personalized feedback on participants’ website use and encourage PA tracking via website features. The tracking feature for the Enhanced group provides adaptive goal setting (e.g., 20% increase of participant’s previous month’s PA) and challenges with opportunities to win tokens on home page (such as the “Golden Tennis Shoe Token” for logging a 10% increase in their daily PA). There is also an online video collection with full-length (10–30 min of MVPA) instructional videos that teach participants the skills necessary to perform PA.

Finally, the discussion board for the Enhanced group is also used to facilitate community meetups among participants to increase social support. Study staff post details of free and low-cost PA events where participants can meet, and promote discussion on coordinating attendance with others, overcoming barriers, and motivating participants to meet at these events. Participants have access to the community board throughout the 24 months of the study.

#### COVID-19-related adaptations

Pasos is a technology-based PA intervention in which most intervention content is delivered remotely via technology channels. Nevertheless, some intervention activities (specifically, randomization and goal-setting sessions at baseline and follow-up) take place during in-person visits. Given social distancing measures during the COVID-19 pandemic, many intervention visits will not take place in person, as originally planned. Thus, our plans for intervention delivery have undergone some adaptations

- Originally, randomization and goal-setting sessions (at baseline and follow-up visits) took place in person at the study site. If a participant was unable to attend an in-person follow-up goal-setting visit, the protocol stipulated completing the goal-setting session over the telephone. As part of the COVID-19-related adaptations, randomization and baseline and follow-up goal-setting sessions take place solely over the telephone and not in person.
- As part of the adaptations, all materials needed for the randomization session are mailed to participants in advance.
- Originally, during randomization visits, intervention staff helped participants to set up their pedometers (which involves determining an individual’s stride). As part of COVID-19-related adaptations, participants are instead provided with step-by-step instructions on how to determine their stride and how to set up their pedometer and are asked to set up their pedometer on their own.

### Criteria for discontinuing or modifying allocated interventions {11b}

N/A: This trial is low risk and we do not expect to need to discontinue the intervention or modify allocation for any given participant.

### Strategies to improve adherence to interventions {11c}

To increase adherence, participants in both conditions will receive brief phone calls at various timepoints to answer questions and direct them back to the website. Participants in the Enhanced group also receive text messages prompting them to return to the website, along with points for logging into the website and using website features. All participants receive incentives for filling out monthly questionnaires ($10/month) to generate the tailored website content.

### Relevant concomitant care permitted or prohibited during the trial {11d}

While there is no concomitant care explicitly permitted or prohibited during the trial, as part of screening procedures participants are asked to report their participation in other clinical trials and investigations; if they have participated in a weight loss or exercise study in the past 6 months, then they are found ineligible to participate in the study.

### Provisions for post-trial care {30}

N/A: Because this trial is low risk and we do not expect any long-term negative effects of the intervention, we have not made provisions for post-trial care.

### Outcomes {12}

The primary outcome of this study is change in mean (or median, if skewed) accelerometer-measured hours/week of moderate-to-vigorous physical activity (MVPA), studied longitudinally (time points: baseline, 6 months, 12 months, 18 months, and 24 months). Additionally, a secondary outcome is change in self-reported MVPA measured via the 7-Day PAR (further described under *measures* below). MVPA has been strongly associated with reduced risk of many adverse health outcomes in adults, including all-cause mortality, cardiovascular disease (heart disease and stroke), hypertension, type 2 diabetes, adverse blood lipid profile, weight gain, cancers (of the bladder, breast, colon, endometrium, esophagus, kidney, lung, and stomach), dementia, depression, and falls (in older adults), as well as improvements in cognition, quality of life, anxiety, sleep, bone health, and physical function [5].

As an exploratory outcome, we will study the differences (among both study arms) in cost per minute increases in weekly mean MVPA, in US dollars. This cost-effectiveness outcome will be studied at 6 and 12 months.

### Participant timeline {13}

**Fig. 1:**
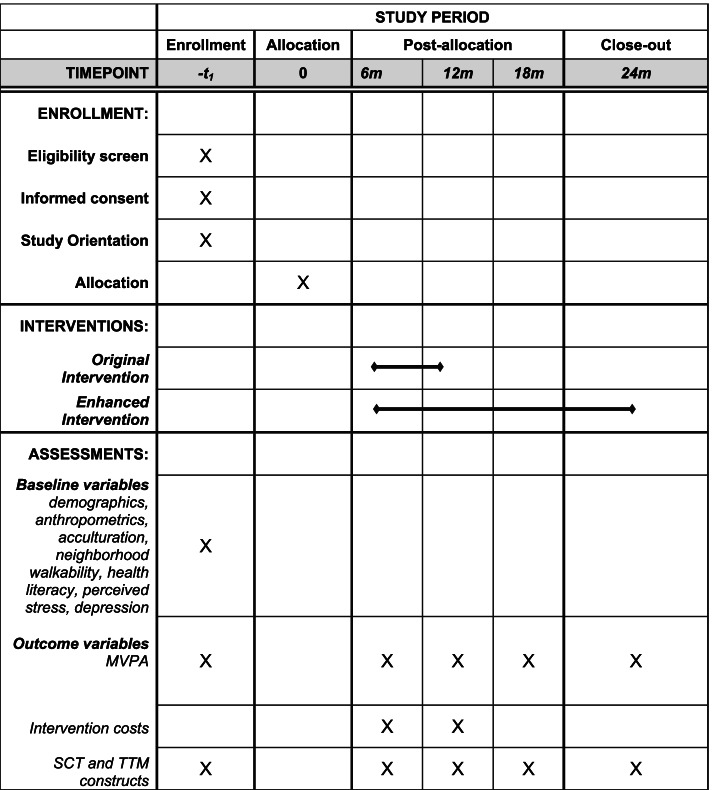
Schedule of enrollment, interventions, and assessments

Participants will initially complete an eligibility screening interview, attend an orientation to learn more about the study, and undergo the informed consent process. At a baseline assessment/randomization, participants will be administered baseline measures and randomly assigned to one of the two (original or enhanced) tailored PA interventions. PA, related theoretical constructs, and intervention costs are assessed again at 6, 12, 18, and 24 months. See Fig. 1.

### Sample size {14}

Our sample size calculation, which yielded a sample size of *N*=300, was conservative and based on the assumption that we would have more than sufficient power (> 80%) for testing the null hypothesis that the intention to treat effect was zero, versus the two-sided alternative that the intervention effect on objectively measured MVPA would be different for those randomized to the enhanced vs. original intervention. Effect size estimates were based on mean objectively measured MVPA at 6 months (75.8, SD=91.0) among those randomized to the Intervention condition in the original Pasos study [10]. We hypothesize that the additions to the enhanced intervention arm would translate into an additional 30 min/week of MVPA at 6 months compared to the original intervention and, as in the original study, these effects would be maintained at follow-up. Given the dose-effect findings from the original study with respect to association between website features and MVPA and the focus on enhancing such features, we were confident that a 30 min/week difference between intervention groups would be a reasonable (if not conservative) assumption. Thus, with 150 participants randomized to each arm at baseline, we would have more than sufficient power (>80%) to detect between-group differences in mean MVPA at follow-ups using a two-tailed significance level *α*=0.05. In addition, given a similar assumption of an additional 30 min of self-reported MVPA among enhanced intervention participants relative to original intervention (112.8 (SD=97.1) in the original study) the proposed sample size would also yield sufficient power (>80%) to test differences in self-reported MVPA. It is important to note that this sample size was deliberately conservative as it did not assume the availability of repeated outcome measures that would be taken throughout the study. By choosing models that make use of the longitudinal data, we would be increasing the power to detect differences between treatment arms.

#### COVID-19-related adaptations

We stopped recruitment when COVID-19 social distancing measures took effect. An amended power calculation was done, and we determined that with a sample size of *N*=210, given effect sizes in the proposed range (>30 min/week difference in MVPA at follow-up between conditions), 20% correlation between covariates in an adjusted model and a two-sided alpha of .05, we would have 81% power for our primary study aim.

### Recruitment {15}

Participants are recruited using community-based strategies that were highly successful in the research team's previous studies with Latinos [9, 21]. These strategies include the use of a Facebook study page, in-person recruiting at community events, advertisements in local Spanish language newspapers and radio, Craigslist advertisements, outreach to Latino-serving community organizations, and posting flyers in the community (e.g., libraries, bodegas/food markets, bus stops, festivals). Additional recruitment strategies include a television advertisement and radio interviews featuring Latina research staff members that aired on Spanish-language networks. Participants are recruited in and near Providence County, Rhode Island, which has the highest concentration of Latinos (24.3%) in the state [22].

## Assignment of interventions: allocation

### Sequence generation {16a}

Participants are randomly assigned to one of the two Spanish language individually tailored Internet-based PA intervention conditions based on a randomization sequence generated using a permuted block randomization scheme, stratified by stage of change. Block sizes are small (2–6) and random and generated using R (www.R-project.org) [23].

### Concealment mechanism {16b}

The project director creates sealed opaque randomization envelopes based on the allocation sequence. Study staff who provide the group allocation to the participants do not have access to the randomization sequence and are not involved in creating the sealed envelopes.

#### COVID-19-related adaptation

Participants randomized in person opened their own randomization envelope, at which point both the participant and the intervention staff would learn the group assignment. As part of the adaptations for remote randomization, the interventionist opened the envelope prior to the randomization session, which was conducted via telephone, in order to mail the appropriate randomization materials to the participant.

### Implementation {16c}

The allocation sequence was generated by the study biostatistician and given to the project director to create sealed randomization envelopes. Study assessment staff enrolls participants and conducts measurement visits. Study intervention staff assigns each participant to an intervention arm by opening one of the sealed envelopes.

## Assignment of interventions: Blinding

### Who will be blinded {17a}

After assignment to interventions, all study outcome assessment staff will remain blinded. Interventionists and study participants are unblinded. We maintain blinding for outcome assessment staff by not involving them in any intervention tasks that would reveal condition assignment and by discussing intervention-related content separately. Additionally, outcome assessment staff do not access sections of the database that would reveal condition assignment.

### Procedure for unblinding if needed {17b}

Participants and intervention staff do not remain blinded after assignment to interventions, and we do not foresee circumstances under which outcome assessment staff need to be unblinded.

## Data collection and management

### Plans for assessment and collection of outcomes {18a}

#### Original plans for assessment and collection of outcomes

##### Baseline assessments

At a baseline measurement visit, participants complete study questionnaires (paper-based) and have measurements taken by study staff (height and weight, blood pressure, waist circumference, percent body fat). At the end of this visit, participants are given an accelerometer and instructions on a 7-day wear protocol. Participants return the accelerometer one week later at their baseline assessment/randomization visit. Wear time is checked using established protocols from our previous trials. If there is insufficient wear time, participants are asked to re-wear the accelerometer and the visit is rescheduled. During this visit, participants also complete the 7-Day Physical Activity Recall interview (7-day PAR, described below) [24, 25]; to improve accuracy of recall and demonstrate moderate-intensity PA, they first participate in a 10-min treadmill walk (3–4 mph) and were asked to refer to the rate of perceived exertion (RPE) scale [26] throughout the walk.

##### Assessments at 6, 12, 18, and 24 months

Follow-up assessment visits take place at 6, 12, 18, and 24 months. Approximately 1 week prior to each scheduled follow-up assessment visit, participants are mailed a questionnaire package and accelerometers to wear again for 7 days, and then return the device and completed questionnaires at the visit. At each follow-up assessment, participants again complete anthropometric and blood pressure measures, the treadmill walk, and the 7-Day PAR interviews. At 12- and 24-month follow-up visits, a Consumer Satisfaction Measure that assesses participant satisfaction with intervention enhancements is administered to all participants. Semi-structured, qualitative interviews with a randomly selected subsample of participants are also completed at 24 months to explore participants’ (1) overall perceptions and satisfaction with the intervention, (2) opinions regarding specific intervention features, particularly the enhancements, (3) suggestions for improving the intervention (how to keep participants logging on to the website and engaged over 24 months), and (4) barriers and facilitators to participating in a clinical trial and staying active over the 2-year project period.

#### Measures

All measures listed below are given at baseline, 6, 12, 18, and 24 at the research center (except where noted), are available in Spanish, and have been used in our previous studies [9–11, 27, 28]. In addition to the measures listed below, the Short Test of Functional Health Literacy in Adults (STOHFLA), is used as an eligibility measure at baseline to determine whether participants have adequate literacy levels to read study materials in Spanish [17, 18, 29]. This measure has high internal validity (>0.95 in either language) and strong positive associations with education. To be eligible, participants needed to score more than 16 on the STOHFLA.

##### Primary outcome

The primary outcome measure is total PA as measured by accelerometer (ActiGraph wGT3X-BT), which measures movement and intensity of PA and has been validated against heart rate telemetry [30, 31] and total energy expenditure [30]. At the end of the Measurement visit, eligible participants receive an Actigraph wGT3X-BT accelerometer, with instructions to wear the accelerometer on their waist with a velcro belt during waking hours for 7 consecutive days. They also receive a form to write down the dates and times they put on and take off the accelerometer. Participants return the accelerometer at the next visit. Per standard procedures, the minimum acceptable wear time is 5 days with at least 600 min daily or 4 days with at least 3000 min total. Daily and weekly minutes of MVPA are calculated with Actilife software, using a minimum cut point of 1952 [31] to define moderate-intensity PA and a minimum activity bout of 10 min, for consistency with the original Pasos Hacia La Salud study [9, 10].

A self-reported measure of total weekly PA is also included as a PA outcome measure. The 7-Day PAR Interview [24, 25] provides an estimate of the total weekly minutes of PA. The 7-Day PAR has been used across many PA studies and our team has extensive experience with administering this instrument, which requires annual recertification. All staff members who administer the 7-Day PAR first undergo in-depth training comprising recorded and scored practice interviews, including various pre-determined practice scenarios. To administer the interview, an interviewer asks participants about moderate, hard, and very hard activities that they might have engaged in during each morning, afternoon, and evening over the past week. The 7-Day PAR has repeatedly shown acceptable internal consistency, reliability, and concurrent validity with objective measures of PA [32–36], along with sensitivity to changes [33, 34] in both moderate and intensive levels of PA [35, 36]. Additionally, the 7-Day PAR has demonstrated test-retest reliability among Latino participants [37].

##### Potential moderators

Potential moderators of intervention effect include demographics, acculturation, neighborhood environment, perceived stress, and depression and are administered at baseline assessment only. Demographic questions assess participant age, education, race, ethnicity, income, employment status, marital status, household size, country of birth, Hispanic subgroup and years lived in the USA. Additionally, the Brief Acculturation Scale (BASH) [38] asks four questions about languages used in different life contexts and has shown good internal consistency in prior research [39].

The Neighborhood Environment Walkability Scale, Abbreviated (NEWS-A) assesses various aspects of the built environment related to walking, neighborhood esthetics, and traffic [34]. It has shown adequate levels of factorial and criterion validity [40]. The Environmental Access questionnaire was also used as a measure of the built environment [41]. The Perceived Stress Scale (PSS) is a widely-used, validated instrument composed of 10 items to measure perceived stress in the past month, with a 5-point scale where 0 means never and 4 means very often [42, 43]. The PSS is well validated and has been used in many studies examining the association between stress and health [42, 43]. The Center for Epidemiological Studies Depression Scale (CES-D) assesses depressive symptoms and has acceptable internal consistencies of 0.87 and above in both English and Spanish [44–46].

Anthropometric Measures include body weight measured by the Health-O-meter medical scale (to the quarter pound) and height measured with a stadiometer (to the quarter inch). An estimate of participant percent body fat is measured using the Quantum II bioelectrical body composition analyzer (RJL Systems, Inc., Detroit, MI). Blood Pressure is measured using a mercury manometer in a sitting position.

##### Potential mediators

Potential mediators of intervention effects include constructs from social cognitive theory and the transtheoretical model. These constructs are assessed at all time points as potential mediators, as well as online each month to generate tailored reports. Constructs include processes of change, self-efficacy, social support, and enjoyment [47, 48]. The 5-item measure of Stages of Change for Physical Activity determines whether a participant is in the Pre-contemplation, Contemplation, Preparation, Action, or Maintenance stage of PA change. This measure has demonstrated reliability and concurrent validity acceptable reliability (Kappa = 0.78; intraclass correlation *r* = 0.84) with measures of self-efficacy and current activity levels [47, 48]. Baseline stage of change will also be examined as a potential moderator of the intervention-PA relationship.

The 40-item Processes of Change for Physical Activity measure asks participants how often (never, seldom, occasionally, often, or repeatedly) they engage in various cognitive and behavioral strategies associated with behavior change [48]. The measure contains 5 behavioral sub-scales (counter-conditioning, helping relationships, reinforcement management, self-liberation, and stimulus control) and 5 cognitive sub-scales (consciousness raising, dramatic relief, environmental re-evaluation, self-re-evaluation, and social liberation). In prior research, internal consistency of the subscales ranged from .62 to .96 [48].

A 5-item Self-Efficacy for Physical Activity measure was included to assess confidence in one’s ability to exercise in various situations on a 5-point scale, ranging from not at all confident to extremely confident [47]. The internal consistency shown in prior research was acceptable (alpha = .82) [47]. The Social Support for Exercise [49] questionnaire measures PA support from three subscales (Family, Friends, Rewards/Punishments) with response options including none, rarely, a few times, often, very often, or does not apply [49]. This measure has shown acceptable internal consistency (alphas .61–.91) and criterion validity [49]. The Physical Activity Enjoyment Scale (PACES) [50] measures the level of enjoyment that a person derives from engaging in PA. Using a scale of 1 to 7, participants are asked to rate their feelings on 18 items about their enjoyment of PA (e.g., a rating of 1 means “I find it pleasurable” and a rating of 7 means “I find it unpleasurable”). The measure has shown high internal consistency (alpha = 0.96) and test-retest reliability [50].

#### COVID-19-related adaptations

Given social distancing measures during the COVID-19 pandemic, many assessment visits will not take place in person, as originally planned. Thus, our plans for assessment and collection of data have undergone various adaptations:

- During the assessment/randomization in-person visit, and during subsequent follow-up visits at 6, 12, 18, and 24 months, participants are asked to complete a 10-min treadmill walk to demonstrate moderate-intensity PA, referencing the RPE scale throughout the walk, to help them to respond to the 7-Day PAR questionnaire. As part of the adaptations, the treadmill walk has been dropped. Participants now thoroughly review the RPE scale with the assistance of assessment staff, to understand the different levels of exertion.
- The stages of change survey were originally completed online during the in-person baseline assessment/randomization visit. As part of the adaptations, this questionnaire is now completed remotely during a telephone call with the assessment staff.
- Originally, a psychosocial questionnaire packet was mailed to participants for completion prior to their baseline and follow-up assessment visits, and participants were asked to bring the completed questionnaires to the visits. As part of the adaptations, we implemented an online version of the psychosocial surveys. Participants are now given the option to complete the questionnaire packet and send it back via mail or complete the questionnaires online.
- As part of the adaptations, measures of height and weight are now collected via self-report. Additionally, data on hip and waist circumference, blood pressure, and body composition are not collected anymore.

### Plans to promote participant retention and complete follow-up {18b}

To promote retention and complete follow-up in the current study, we will use strategies that have been successful for retaining Latina participants in our prior trials [10, 27, 28, 51]. For example, the intervention will be offered in Spanish at no cost over the Internet. Bilingual/bicultural study staff will provide flexible scheduling and reminders for assessment appointments, along with financial incentives and reimbursement for related travel and childcare costs. Outcome data will be collected from all participants, including those who discontinue or deviate from the intervention protocols and are still willing to participate in assessments.

### Data management {19}

Data management is provided by an experience Data Systems Analyst, who designs all databases and coordinates data entry, verification, auditing, and programming of the interface between data entry systems. Data is collected via paper questionnaires and entered into the study database by assessment study staff, or collected via online questionnaires and directly transferred into the study database. For security and confidentiality purposes, all study data is coded with participant ID numbers, and documents with identifying information are masked and stored separately, as described in the confidentiality section below.

All data designated as primary outcome data in the grant proposal will be subject to a 100% cross-referencing with the original paper copy. This audit must have an error rate of less than 1%. If the verification fails the audit, all data will be re-entered, the original computer files discarded, and the newly re-entered data audited. This process will continue until the audit no longer exceeds the maximum allowable error rate.

All other entered information (non-primary outcome data) will be subject to a 20% sample that will be cross-referenced with the original paper copy. This audit must have an error rate of less than 1%. If the sample fails the audit, all data will be verified against the paper originals. If the error rate of the complete audit is greater than 1% then all data will be re-entered, the original computer files discarded, and the newly re-entered data audited. This process will continue until the audit no longer exceeds the maximum allowable error rate. At the discretion of the study’s Principal Investigator, the full audit may be omitted in favor of a complete re-entry of the original paper data. All audits will be supervised and documented by the study’s Data Systems Analyst.

### Confidentiality {27}

To protect the confidentiality of participant data, the team will assign code numbers to all data. Any documents with identifying information (e.g., consent forms, participant name, and contact information) will be kept in a separate locked cabinet from participant data. Study materials will be stored in a locked filing cabinet at Brown University and on password-protected software in a secure office. Only the study team will have access to this information. The de-identified data will be stored in a password-protected location for the duration of data analysis. All research staff will receive ethics training and certification through Brown University and there will be regular team meetings to ensure all members of the team are informed and following the protocol to protect confidentiality of participant records. Staff are required to sign a confidentiality oath and are informed about the importance of and the definitions of confidentiality. Any breach of confidentiality will result in disciplinary action up to and including termination of employment as per university policies. All breaches of confidentiality will be reported by the principal investigator to the Institutional Review Board within 24 h.

All subject-identifying information will be destroyed after recruitment and analyses are completed for long-term storage of the data sets. Strict confidentiality procedures will be in place for circumstances where staff will need access to subject’s names. However, once the intervention is completed, staff will no longer have access to that participant data.

### Plans for collection, laboratory evaluation, and storage of biological specimens for genetic or molecular analysis in this trial/future use {33}

N/A: No biological specimens will be collected in this trial.

## Statistical methods

### Statistical methods for primary and secondary outcomes {20a}

As a preliminary step, we will assess potential between-group differences in baseline characteristics (demographics, activity level, psychosocial constructs) using graphical methods, non-parametric and parametric tests as appropriate (e.g., Wilcoxon rank-sum test for skewed data, t-tests for normally distributed continuous data & chi-squared tests for categorical data). Any variables not balanced by randomization will be controlled for as covariates in subsequent analyses. In addition, website use will be summarized (e.g., number of logins per month, average time spent on each website) and compared between-groups using chi-squared tests (binary measures) and t-tests (continuous measures).

Consistent with our primary aims, we will examine the effects of the enhanced vs. the original intervention on our *primary outcome*: objectively measured MVPA over 24 months. To avoid the effect of outliers, we will apply a normalizing transformation (if necessary) to the outcome (*Y*_*i,j*_=MVPA for participant *i* at follow-up *j*) prior to analysis. A single linear mixed effects regression model will be used to simultaneously estimate the intervention effects on outcome (*i*=1,2,…,210, *j*=1,2,3,4 where j indexes follow-ups at months 6, 12, 18, and 24) with a subject-specific intercept included to account for within-subject correlation in the outcome over time. Data are nested such that weekly MVPA is nested within a month within participant and standard errors must be corrected for such a design consideration. We will control for the baseline value of the outcome and potential confounders of the treatment effect (including any variables not equally distributed by randomization, as well as a time-varying indicator of pre vs during pandemic time). Mixed effects models use a likelihood-based approach to estimation and therefore make use of all available data (intent-to-treat sample) without directly imputing missing values. A similar series of regression models will be used to assess between-group differences in the secondary measure (self-reported MVPA). Should the final outcomes data be skewed and efforts to bring towards normality not successful, a longitudinal quantile regression model will be used to assess group effects on median min/week of MVPA (instead of mean min/week).

### Interim analyses {21b}

N/A: No interim analyses are planned or have been conducted.

### Methods for additional analyses (e.g. subgroup analyses) {20b}

We will investigate potential mediators of the intervention effect (e.g., self-efficacy, enjoyment, social support) using a multiple mediation approach, in which all potential mediators are tested simultaneously, using a product of coefficients method [52] with bootstrapped standard errors (5000 samples with replacement). We will estimate the path coefficients (*a* path: effects of the intervention on changes in mediators from baseline to end-of-treatment and *b* path: effects of changes in the mediators on MVPA at 6, 12, 18, and 24 months follow-ups, controlling for baseline), as well as the indirect effect of the intervention (*ab* path: effect of the intervention on MVPA through the mediators). Interest is in estimating the path coefficients, effect sizes, and confidence intervals, rather than strict hypothesis testing.

Additionally, a variable will be considered a moderator if evidence exists of either qualitative or quantitative interaction with the intervention. We will use a similar analytic approach to that described for the Primary Aims; models will include the main effects of intervention (enhanced vs. original), the potential moderator (e.g., acculturation), as well as the interaction between the two. Evidence of moderation exists if the coefficient of the interaction term is statistically different than zero. Significant moderators will be further examined using subgroup analyses.

To determine the relative costs of delivering the original vs. enhanced intervention, we will be tracking the costs associated with program delivery and the costs associated with research. Cost collection will identify intervention delivery costs as well as costs to participants (including opportunity costs) of the intervention and of devoting time to PA. Our analysis will allow for an examination of factors that can be changed to improve efficiency of future intervention development and provide input data that will permit simulation modeling of real world-implementation costs and effects. Cost data collection will follow generally accepted practice in cost-effectiveness research [53, 54]. In addition, the cost collection procedures and estimations will borrow from methods derived from process engineering and managerial accounting. The cost tracking system will track resources used to deliver the intervention as well as identify research protocol-driven costs that would not be part of a real-world implementation [55]. A time tracking system will be used to identify the time utilization of intervention staff devoted to the intervention. Research-related activities unrelated to the intervention delivery (e.g., obtaining consent) will also be tracked and removed from the analysis of intervention costs.

The current research team has experience with developing time tracking systems that capture information at the clinician and client level without encumbering the clinical process (e.g., HL64342). The cost tracking system will identify intervention development costs as well as operational costs associated with running the intervention. Some of the operational costs will include personnel costs, Internet, telephone, and facility costs (e.g., administrative support). We will also track the cost of developing new technological features, such as the enhanced website and text messaging system, as well as the staff time needed to implement these features into the respective intervention arms. Technology costs will be sourced directly from invoices from Illumina, Inc., which developed the website for the original Pasos study. Staff time and other resources used at the participant level will be tracked to identify variations in service delivery and cost-efficiency across participants. Incremental cost-effectiveness ratios between conditions will be calculated as the differences in costs per minute increase in weekly physical activity. Our team has also developed expedient methods for assessing participant costs through questionnaires. These assessments include identification of time devoted to the intervention, time spent in PA, incremental out-of-pocket expenses for PA, variation in productivity (i.e., work absenteeism), and medical office visits for activity-related injuries. Since the cost analysis will focus on the provider perspective as the main decision-maker and the perspective of participants, other societal costs (e.g., medical costs) are not included in this preliminary analysis. As better data become available through epidemiological and health services studies of these latter costs, we will be able to account for these in our modeling.

### Methods in analysis to handle protocol non-adherence and any statistical methods to handle missing data {20c}

We will apply two statistical approaches to handling missing data and compare these to the effect estimates from the primary outcomes’ analysis (which take a likelihood-based approach to estimation but do not directly impute missing data). The first is inverse probability weighting with propensity scores. This is a two-step method: first, we will model the probability of missingness as a function of baseline covariates and previous outcomes (using logistic regression). The resulting predicted probabilities of dropout will serve as the propensity scores. Next, the inverse propensity scores serve as weights in our regression model of the primary outcomes (i.e., MVPA). Provided the data are missing at random (MAR) or that the probability of missingness can be fully explained by observable data, this approach produces asymptotically unbiased estimates. To allow for the possibility that the MAR assumption may not hold, we also will use pattern mixture models. In this case, the distribution of the primary outcome is assumed to follow a mixture of two distributions: one for those who complete follow-up and another for those who do not. This method allows us to quantify the robustness of the study findings to a range of missing data assumptions.

### Plans to give access to the full protocol, participant-level data and statistical code {31c}

We plan to grant access to participant-level dataset and statistical code by request only.

## Oversight and monitoring

### Composition of the coordinating center and trial steering committee {5d}

This study is a single-site study. The team that provides organizational support and runs the trial day-to-day meets on a weekly basis and is composed of a project director and two research assistants, one of whom focuses on all assessment-related tasks, while the other focuses on all intervention-related tasks. The project director coordinates all aspects of the project, serves as the liaison between the intervention staff and the evaluation staff, coordinates tracking information related to exploratory cost analyses, and monitors the quality of data collected at all time points (by supervising and responding to any questions the research assistants have regarding data collection).

Additionally, the project director, research assistants, the principal investigator, and co-investigators meet on a weekly basis to discuss new research developments, make decisions regarding changes to the research protocol or to specific research activities, and to problem-solve if necessary.

The larger team of investigators (which includes the principal investigator, co-investigators, investigators on subawards, and consultants) meets on a quarterly basis to discuss study progress and challenges faced; problem solve, review and approve changes to study procedures; and manuscript preparation and progress.

Data management is provided by an experienced data systems analyst, who works with the project director to train personnel (on an as-needed basis), to code the data and run the computer systems. The data systems analyst also works with the study statistician throughout the trial to ensure all data is entered, stored, and ready for analysis.

### Composition of the data monitoring committee, its role and reporting structure {21a}

Because this study is minimal risk and single site, the principal investigator (Dr. Bess Marcus) will ultimately be responsible for monitoring and overseeing participant safety and adverse events. In addition, there will be a team that will help with the reporting and monitoring process. This team, which is independent from the sponsor (the National Cancer Institute), will comprise the principal investigator (Dr. Marcus), the project director, and the study physician. The project director will be responsible for communicating with participants who report injuries and alerting the other members of the monitoring team about the adverse event or unanticipated problem. The study physician will be responsible for identifying the proper procedure (e.g., whether medical care is necessary) based on the severity of the injury, and will also communicate directly with injured participants to assess their symptoms. Dr. Marcus will be responsible for the reporting of adverse events or unanticipated problems and submitting any necessary reports to the Institutional Review Board and the National Cancer Institute. The project director will also review the Health Expense Forms that participants complete every month to monitor any injuries that may have occurred because of, or in relation to, exercise training, and any costs associated with them, and will discuss these with the principal investigator and study physician.

### Adverse event reporting and harms {22}

Potential complications associated with moderate-intensity exercise training at levels recommended by the Centers for Disease Control and Prevention and the American College of Sports Medicine are rare. However, subjects may find exercising uncomfortable and may experience sprains, other soft tissue injuries, or bone injuries. At the start of the study, all participants will be given an instruction sheet that details what to do for common exercise-related injuries, as well as the project director's contact information. Participants will be asked to notify us immediately (no later than 24 h) if they sustain an injury. In addition, we will require each participant to complete a monthly Health Expense Form . For any adverse event spontaneously reported to the project director or identified on the monthly Health Expense Form (i.e., solicited), the monitoring team will meet and discuss within 24 h of the participant reporting.

The monitoring team will consider the following three guidelines for reporting of adverse events: (1) Is the adverse event unexpected in nature, severity, or frequency?; (2) Is the adverse event related or possibly related to participation in the research?; and (3) Does the adverse event suggest that the research places subjects or others at greater risk of physical or psychological harm than was previously known or recognized? If none of the questions are found to be true, the adverse event is not an unexpected or serious adverse event, but an expected adverse event. Such events will be reported to the Institutional Review Board (IRB) and the National Cancer Institute (NCI) as part of our annual report. If the adverse event falls outside of the potential risks described in the protocol and consent documents, it is classified as an unanticipated adverse event. If the unanticipated problem results in death, is life-threatening, results in inpatient hospitalization, results in persistent or significant disability or requires medical intervention to prevent any of these outcomes, it will be classified as a serious adverse event. In the case of a serious and/or unanticipated adverse event, the principal investigator will immediately (no later than 24 h) report it to the Brown University Institutional Review Board and to NCI, along with a description of any changes to the protocol or other corrective actions that have been taken or are proposed in response.

### Frequency and plans for auditing trial conduct {23}

Quarterly quality assurance checks will be completed to verify compliance with study procedures and IRB requirements. Specifically, staff will verify that all consent forms and other protected health information are secured and accounted for. In addition, on a quarterly basis, staff members will participate in a training reviewing the procedures for handling emergency situations that may arise during in-person visits.

### Plans for communicating important protocol amendments to relevant parties (e.g. trial participants, ethical committees) {25}

Any changes to the study protocol will be submitted for approval by Institutional Review Board before their implementation using the Institutional Review Board's amendment request forms. Any major changes to the protocol that may affect participants’ activities and involvement will be communicated via posts on the message board on the Pasos website and via emails.

### Dissemination plans {31a}

We will communicate our findings to other PA researchers during the American College of Sports Medicine, and the Society for Behavioral Medicine annual meeting. Additionally, to communicate our findings to the public and to healthcare professionals, we plan to publish findings in top journals in public health, medicine, and prevention, including the American Journal of Preventive Medicine, Preventive Medicine, and Health Psychology. In addition, all investigators have wide networks of contacts within and outside of academia and will disseminate findings accordingly.

## Discussion

Given the high rates of inactivity and related co-morbidities among Latina women [3, 4, 6], this is a priority population for PA promotion. Efficacious and cost-effective PA interventions for Latina women are urgently needed to address this public health concern and advance health equity. The Pasos Hacia La Salud II RCT aims to test the efficacy of a theory- and technology-enhanced PA intervention for increasing and maintaining minutes of MVPA among Latina women, compared to an original version of the intervention. Additionally, cost-effectiveness and potential mediators and moderators of the intervention effect on MVPA will be evaluated. The original Pasos PA intervention showed efficacy in helping Latinas increase PA. We expect the additions in the enhanced intervention to help a larger proportion of participants to increase and maintain their PA long term (i.e., 24 months) as well as to reach national guidelines of 150 min of MVPA per week. Moreover, the web- and text-based enhanced intervention could have great reach and dissemination potential, which could be capitalized on in the future to help to advance health equity.

Among study strengths is the use of evidence-based intervention and assessment strategies, which are guided by prior efficacy testing [10, 11, 56] and extensive formative research with Latinas [9] and grounded in strong behavioral science theory and thus likely to result in improved health outcomes. Moreover, the use of Internet and mediated technologies for intervention delivery supports future widespread dissemination of the intervention once efficacy has been satisfactorily evaluated. Indeed, a future research direction entails the use of Dissemination and Implementation Science to explore the dissemination and implementation of this evidence-based intervention through primary care and community-based networks.

Alternatively, among study limitations, is the requirement that participants have access to the Internet and to a cell phone as an eligibility criterion. While Latinos in the USA now have nearly equal Internet and mobile phone access as non-Hispanic whites [8], this requirement may reduce the generalizability of findings. Moreover, the intervention is only offered in Spanish, which may exclude more acculturated Latina women who may prefer to receive intervention materials in English. Future studies could offer the intervention in both Spanish and English for increased reach and generalizability.

While Pasos was originally conceived as a technology-based intervention with many components delivered remotely, some components of the intervention and the study assessments that were originally planned as in-person activities had to be adapted in response to the COVID-19 pandemic and ensuing social distancing measures. We have aimed to maintain the highest degree of fidelity that is possible. In this manuscript, we aimed to report the original study protocol for the Pasos Hacia La Salud RCT, as well as the various adaptations made throughout. We decided to submit this manuscript for review only after all necessary adaptations were completed, in order to reflect and include all pertinent information. It is our hope that our description of both the original protocol and the adaptations made in response to the pandemic will be helpful to other researchers and will inform future interventions. We also believe that the resulting fully-remote version of the Pasos Hacia La Salud II study strengthens our capability for future dissemination and implementation of the PA intervention in real-world settings.

## Trial status

Active, not recruiting. Recruitment for this trial began in April 2018 and was completed in April 2020.

## Data Availability

The final trial dataset will be made available upon request.

## Abbreviations

PA: Physical activity
MVPA: Moderate-to-vigorous physical activity
7-Day PAR: 7-day Physical Activity Recall
RCT: Randomized controlled trial
SCT: Social cognitive theory
TTM: Transtheoretical model
RPE: Rate of perceived exertion
STOHFLA: Short Test of Functional Health Literacy in Adults
BASH: Brief Acculturation Scale
NEWS-A: Neighborhood Environment Walkability Scale, Abbreviated
PSS: Perceived Stress Scale
CES-D: Center for Epidemiological Studies Depression Scale
PACES: Physical Activity Enjoyment Scale
MAR: Missing at random
IRB: Institutional Review Board
NCI: National Cancer Institute
